# Meta-Analysis of the Prognostic and Predictive Role of the CpG Island Methylator Phenotype in Colorectal Cancer

**DOI:** 10.1155/2022/4254862

**Published:** 2022-09-15

**Authors:** Jing Wang, Zhujun Deng, Xiaoqiang Lang, Juan Jiang, Kang Xie, Sifen Lu, Qiongxia Hu, Yuwei Huo, Xinru Xiong, Niu Zhu, Wengeng Zhang

**Affiliations:** Precision Medicine Center, Precision Medicine Key Laboratory of Sichuan Province, West China Hospital, Sichuan University, Chengdu, Sichuan, China

## Abstract

**Background:**

Various studies have produced contradictory results on the prognostic role of the CpG island methylator phenotype (CIMP) among colorectal cancer (CRC) patients. Although a meta-analysis published in 2014 reported a worse prognosis of CIMP among CIMP-high (CIMP-H) CRC patients, the sample sizes of the major included studies were small. In this study, we included the most recent studies with large sample sizes and performed an updated meta-analysis on the relationship between CIMP and CRC prognosis.

**Methods:**

A search of MEDLINE, Web of Science, and Cochrane for studies related to CIMP and CRC published until July 2021 was conducted based on the PICO (participant, intervention, control, outcome) framework. Data extraction and literature analyses were performed according to PRISMA standards.

**Results:**

In the present update, 36 eligible studies (20 recently published) reported survival data in 15315 CRC patients, 18.3% of whom were characterized as CIMP-H. Pooled analysis suggested that CIMP-H was associated with poorer overall survival (OS) (hazard ratio [HR] = 1.37, 95% CI: 1.26–1.48) and disease-free survival/progression-free survival/recurrence-free survival (DFS/PFS/RFS) (HR = 1.51, 95% CI: 1.19–1.91) among CRC patients. Subgroup analysis based on tumor stage and DNA mismatch repair (MMR) status showed that only patients with stages III-IV and proficient MMR (pMMR) tumors showed a significant association between CIMP-H and shorter OS, with HRs of 1.52 and 1.37, respectively. Three studies were pooled to explore the predictive value of CIMP on CRC patient DFS after receiving postoperative chemotherapy, and no significant correlation was found.

**Conclusion:**

CIMP-H is associated with a significantly poor prognosis in CRC patients, especially those with stage III-IV and pMMR tumors. However, the predictive value of CIMP needs to be confirmed by more prospective randomized studies.

## 1. Introduction

Globally, colorectal cancer (CRC) is the third most commonly diagnosed cancer and the second most common cause of cancer death, with an estimated 1.8 million new cases (10.2% of all cases) and 881,000 cancer deaths (9.2% of the total cancer deaths) in 2018 [1]. CRC is a complex and genetically heterogeneous disease that develops as a result of a series of genetic and epigenetic changes that promote tumorigenesis and metastasis in the intestine [2–4]. Chromosomal instability (CIN), the CpG island methylator phenotype (CIMP), and microsatellite instability (MSI) are three major considerations for CRC development [5].

CIMP CRCs, representing approximately 15% of CRCs, are defined by global genome hypermethylation in CpG islands, which was originally introduced by Toyota in 1999 [5, 6]. CpG islands are usually characterized by the following criteria: DNA sequences greater than 200-500 bases in length, CG base composition higher than 50%, and observed/expected (O/E) CpG ratio greater than or equal to 0.6 [7]. The aberrant methylation of CpG islands in the promoter regions of tumor suppressor genes is correlated with transcriptional silencing, abnormal cell proliferation, oncogenic transformation, and tumor progression [8–10]. Regardless of the biological effect of methylation-induced gene silencing, this DNA methylation alteration pattern has been considered a promising biomarker for CRC prognosis and prediction for years [8, 11–13].

CIMP-high (CIMP-H) CRCs have been linked to poor survival in studies [10, 11, 14–16]. However, more studies observed no correlation between CIMP-H and CRC prognosis [12, 17–26]. To examine whether CIMP status might be used as a prognostic marker for CRC, Juo and colleagues summarized the published studies with controversial findings and conducted a systematic review and meta-analysis for the first time in 2014 [27]. In this review, 11 studies with 3559 patients and 7 studies with 1454 patients provided satisfactory adjusted HR estimates regarding the impacts of CIMP status on survival among CRC patients. Even though the results suggested a worse prognosis among CIMP-H CRC patients with marginal significance (*P* < 0.01), the sample sizes of the major studies included in this review were relatively small. To date, a number of new studies with large sample sizes have explored the connection between CIMP status and CRC prognosis, exhibiting a lack of consistency [28–31]. This suggested that a more comprehensive synthesis of all the relevant data was needed to add weight to the findings of the first meta-analysis. Therefore, the main objective of our research was to conduct an updated meta-analysis by including the most recently published studies to gain further insight into the prognostic efficacy of CIMP status in CRC.

In addition to the impact of CIMP on the prognosis of CRC patients, we also wanted to determine whether CIMP status would affect treatment decisions for CRC patients. Adjuvant chemotherapy based on 5-fluorouracil (5-FU) has been suggested for stage II-III CRC patients after resection for many years [32], and several germline variants may play a role in the response to adjuvant treatment [33]. Rijnsoever et al. reported that the poor prognostic value of CIMP-H was observed only in patients receiving surgery alone but not in patients treated with surgery plus 5-FU chemotherapy [34]. This result implies that CIMP-H CRC patients could benefit from 5-FU-based treatment, but this finding was still controversial in other studies [12, 24, 35, 36]. Thus, a secondary purpose of this research was to determine whether CIMP status might be used as a biomarker for CRC patients undergoing surgery plus chemotherapy.

## 2. Methods

This meta-analysis was registered with Prospero (CRD42021292104), and the PRISMA guidelines were followed for reporting [37]. The PICO (participant, intervention, control, and outcome) criteria were used for literature search, and the PICO characteristics were as follows: (1) CRC patients; (2) the CpG island methylator phenotype of CRC was defined as high (CIMP-H), or low (CIMP-L); (3) the CpG island methylator phenotype of CRC was defined as low and negative (CIMP-L/N), or negative (CIMP-N); and (4) overall survival (OS), disease-free survival (DFS), progression-free survival (PFS), and recurrence-free survival (RFS).

### 2.1. Search Strategy

A thorough literature search was undertaken to discover original English papers published up to July 2021 using three main databases: MEDLINE, Web of Science, and Cochrane. The Medical Subject Heading (MeSH) thesauri used were CpG island methylator phenotype, colorectal neoplasms, survival, prognosis, prognostic, predictive, predictor, and the related free thesauri. Supplementary Appendix 1 shows the PubMed electronic database's specialized search approach. A search for the major references of the included studies was also carried out for eligible articles.

### 2.2. Eligibility Criteria

Published studies reporting the association between CIMP status and CRC survival were included. The main outcomes of interest were OS and DFS/PFS/RFS. The evaluation method of CIMP status should be mentioned in each eligible study. The research design, ethnicity, tumor stage, and sample size were not limited. Studies with the same authors were carefully examined to avoid including duplicated data from the same population. Only peer-reviewed studies were included. Unpublished data, conference abstracts, editorials, notes, letters, review articles, and case reports were excluded.

### 2.3. Data Extraction and Quality Assessment

Two researchers independently extracted data from each eligible study by means of a predefined datasheet. Any doubts or disagreements were settled by consensus or by a third examiner. Data items extracted from each article included the year of publication, first author, continent or country, number of patients, age, follow-up time, treatment modality, tumor stage, CIMP assessment information (CIMP markers, CIMP testing method, threshold of CIMP-H, and CIMP-H prevalence), and relevant survival outcomes.

The quality of each included study was evaluated using the Newcastle–Ottawa Quality Assessment Scale (NOS) (Supplementary Appendix 2) [38]. A total of 9 points based on 3 items were assessed: selection, comparability, and outcome assessment. Only articles with a score of more than 6 could be included in this study.

### 2.4. Statistical Analysis

Data analysis was completed with Review Manager version 5.3 (The Nordic Cochrane Centre, København, Denmark) and/or Stata version 12 (Stata Corp, College Station, TX). We tried to extract and process the raw data from the original literature based on the strategy reported by Tierney et al. [39] when the hazard ratio (HR) and its 95% confidence interval (CI) were not reported. RFS was interpreted as synonymous with DFS. Heterogeneity among different studies was measured with Cochran Q (*P*) and I-square (*I*^2^) tests. *P* < 0.1 or *I*^2^ > 50% indicated substantial heterogeneity. A randomized effect model was used when heterogeneity was significant; otherwise, a fixed effect model was used. The source of the heterogeneity was detected by the Galbraith plot. The existence of publication bias was estimated using a funnel plot and Egger's linear regression test. The stability of the results was checked by sensitivity analysis.

## 3. Results

### 3.1. Literature Search and Study Characteristics

**Figure 1:**
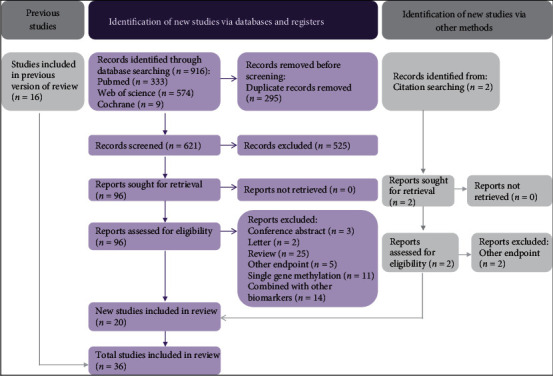
PRISMA 2020 flow chart.

A total of 918 articles were initially identified through the document search. After 295 duplications were removed, 525 records were discarded based on their titles and abstracts. Next, the remaining 98 articles were subjected to a full-text review to determine their eligibility, with 62 records being eliminated (Figure 1). Finally, 36 studies with a quality score of 7 to 9 were eligible for this meta-analysis.

The detailed features of the 36 eligible studies published between 2005 and 2020 are presented in Table 1. There were 15315 patients in these studies, with a median sample size of 263 (range 33-1867), and the majority of them (31 of 36) had more than 100 patients. Among these included studies, 17 studies were conducted in Asia, 11 studies in America, and 8 studies in Europe. Approximately 60% of the studies reported the median follow-up time, ranging from 38 to 112.8 months (median, 58.8 months). Only 16 studies reported details about medication modalities, with the exception of one study that reported anti-EGFR therapy; the other studies were based on either oxaliplatin-based or fluoropyrimidine-based chemotherapy.

### 3.2. CIMP Definition

All studies had clear methodologies for the assessment of CIMP status. With no general consensus, the gene panel, laboratory method, and CIMP-high threshold used to define CIMP varied among studies. The number of methylation markers evaluated for each study varied from 5 to 15, with a median of 5 markers. The Weisenberger panel (CACNA1G, IGF2, NEUROG1, RUNX3, and SOCS1) [40], the classical panel (MINT1, MINT2, MINT31, CKKN2A(p16), and hMLH1), or gene panels that combine these two panels were used in 29 out of the 36 studies. A total of 6 laboratory methods were used to detect gene methylation status. Methylation-specific PCR (MSP) and MethyLight assay (methylation-specific real-time quantitative PCR) were the two most commonly employed methods, whereas only 4 of the 36 included studies chose to use other testing methods. The CIMP was classified by a trichotomy of CIMP-high (CIMP-H), CIMP-low (CIMP-L), and CIMP-negative (CIMP-N) in 5 studies, by a dichotomy of CIMP-H and CIMP-L/N (CIMP-L combined with CIMP-N) in 30 studies, and by both methods in 1 study. The median prevalence of CIMP-H was 18.3% (range, 4.6% to 48.5%).

### 3.3. Overall Survival

**Figure 2:**
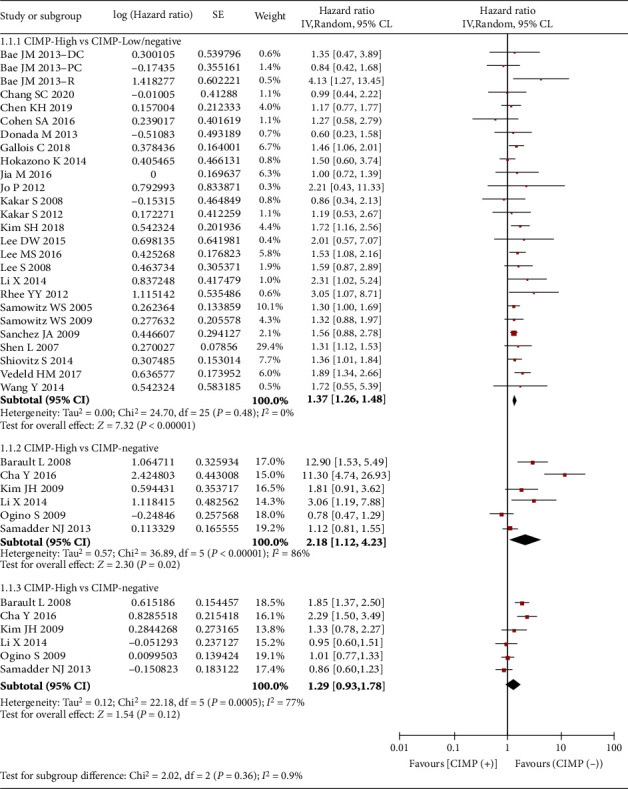
Forest plots of HRs for OS of CRC associated with CIMP. In the dichotomy, CIMP(+) represents CIMP-high, and CIMP(-) represents CIMP-low plus CIMP-negative. In the trichotomy, CIMP(+) represents CIMP-high or CIMP-low, and CIMP(-) represents CIMP-negative.

Twenty-six studies (12930 patients, 2142 CIMP-H) were eligible for pooling data on OS. Twenty-one of the investigations used a dichotomized classification system (CIMP-H versus CIMP-L/N). The summary HR estimate was 1.37 (95% CI: 1.26–1.48), with no obvious statistical heterogeneity (*I*^2^ = 0%, Cochran's Q *P* = 0.48, Figure 2), indicating a shorter OS for patients with CIMP-H CRC. Both the funnel plot (Supplementary Figure 1) and Egger's test (*P* = 0.404) showed no significant publication bias. Sensitivity analysis showed that the overall HR was stable and was not influenced by any individual study (Supplementary Figure 2). Of the 6 other studies classifying CIMP trichotomized (CIMP-H versus CIMP-N or CIMP-L versus CIMP-N), only CIMP-H was associated with substantially worse OS than CIMP-N (summary HR 2.18 with 95% CI 1.12–4.23, Figure 2).

Eleven studies investigated the relationship between CIMP and OS in patients with different tumor stages. Of these, 4 studies with 1073 patients and 10 studies with 4250 patients reported data in stages I-II and stages III-IV CRCs, respectively. Subgroup analysis stratified by tumor stage suggested that CIMP-H was associated with poor OS for stages III-IV CRCs (HR: 1.52, 95% CI: 1.27-1.81, Supplementary Figure 3) but not for stage I-II CRCs (HR: 0.67, 95% CI: 0.42-1.09) compared to CIMP-L/N.

Twelve studies evaluated the correlation between CIMP and OS based on DNA mismatch repair (MMR) status. Of these, 9 studies with 5686 patients and 7 studies with 894 patients presented data on pMMR and dMMR tumors, respectively. In the proficient MMR (pMMR) subgroup, CIMP-H, compared with CIMP-L/N, showed significantly worse OS (HR: 1.37, 95% CI: 1.08-1.75, Supplementary Figure 4). However, no significant difference in OS was found in the deficient MMR (dMMR) subgroup (HR: 1.63, 95% CI: 0.96-2.76).

The overall result suggested a shorter OS for CIMP-H CRC patients, especially those with stage III-IV and pMMR tumors.

### 3.4. Disease-Free Survival/Progression-Free Survival/Recurrence-Free Survival

**Figure 3:**
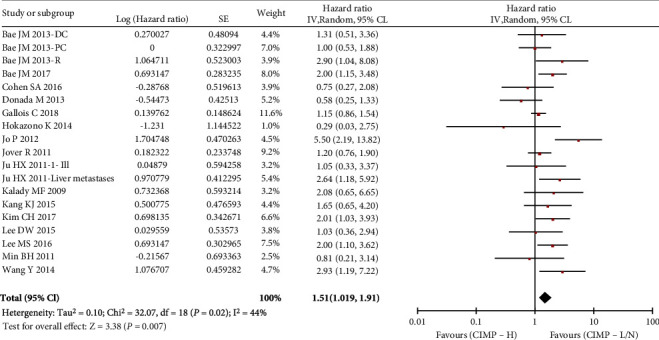
Forest plots of HRs of DFS/PFS/RFS in studies of CRC patients associated with CIMP.

**Figure 4:**
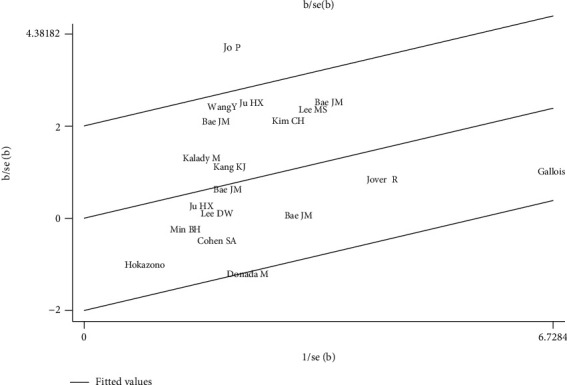
Galbraith plot of the association between CIMP and DFS/PFS/RFS in CRC patients.

Sixteen studies (6142 patients, 828 CIMP-H) were suitable for pooling DFS/PFS/RFS data. All studies classified the CIMP dichotomized (CIMP-H versus CIMP-L/N). The pooled HR for CIMP-H tumors was 1.51 (95% CI: 1.19–1.91), with substantial heterogeneity (*I*^2^ = 44%, Cochran's Q *P* = 0.02, Figure 3). No obvious evidence of publication bias was found by funnel plot (Supplementary Figure 5) or Egger's test (*P* = 0.588). Both the Galbraith plot (Figure 4) and the sensitivity analysis (Supplementary Figure 6) suggested that 1 study reported by Jo P et al. might be the major source for heterogeneity. After removing this study, the heterogeneity was indeed reduced (*I*^2^ = 28%, Cochran's Q *P* = 0.13), but it did not change the orientation of the new joint estimate (HR = 1.37, 95% CI: 1.16-1.61). The results therefore demonstrated unfavorable DFS/PFS/RFS for CIMP-H CRC patients.

**Figure 5:**
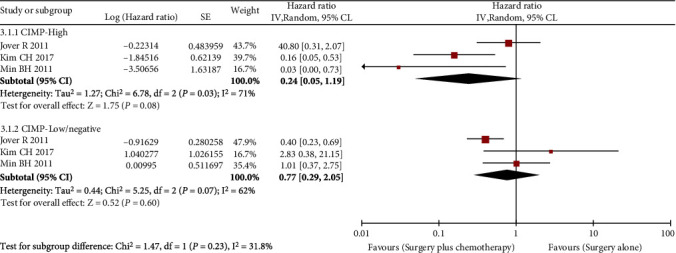
Forest plots of HRs for the effect of chemotherapy on DFS by CIMP status.

Three studies with 433 patients reported the effectiveness of 5-FU-based chemotherapy on DFS by CIMP status. In total, 77 (61%) of 127 CIMP-H patients and 200 (65%) of 306 CIMP-L/N patients underwent chemotherapy following curative resection of the tumor. However, postoperative chemotherapy did not significantly enhance DFS in CIMP-H CRC patients (summary HR = 0.24, 95% CI: 0.05-1.19, Figure 5), nor did it benefit CIMP-L/N patients (summary HR = 0.77, 95% CI: 0.29-2.05, Figure 5).

## 4. Discussion

Alteration of CIMP status has been considered one of the main molecular mechanisms of CRC tumorigenesis for many years. A previous meta-analysis reported that no significant difference was observed for the prevalence of CIMP-H across North and South America, Europe, Australia, and Asia and the pooled prevalence was 22% (95% CI: 18–26%) [41]. Similar to that meta-analysis, almost one in five (18.3%) individuals had CIMP-H CRCs in our study. This suggested that a deeper understanding of the prognostic efficacy of CIMP status in CRC would be helpful in clinical decision making to improve patients' clinical outcomes and care.

To date, one of the biggest challenges is that no universal standard exists regarding the laboratory techniques, gene panels, and marker threshold values for the definition of CIMP-H [42]. In this review, several laboratory techniques for CIMP detection have been used across different studies, including the MethyLight assay, MSP, methylation-sensitive high-resolution melting (MS-HRM), bisulfite pyrosequencing, and Methylation450 bead-chip [28, 36, 43–45]. MSP and MethyLight assays are the simplest and most commonly used methods for qualitatively or quantitatively testing the methylation status of CpG sites in genes. More importantly, using either MSP or MethyLight assays, CIMP-H was consistently associated with an unfavorable prognosis (Supplementary Figure 7). Regarding the CIMP panels, at least 16 different panels have been reported, and no significant difference was observed in the prognostic value [42]. Therefore, despite the heterogeneity of CIMP definitions, the numerical synthesis of different studies to comprehensively analyze the association between CIMP and CRC prognosis is still worthwhile.

The first meta-analysis published in 2014 reported that CIMP-H was associated with a worse outcome for CRC patients [27]. To gain further insight into the prognostic value of CIMP among CRC patients, we conducted an updated meta-analysis. In this manuscript, we identified 20 recently published studies [13, 28–31, 35, 36, 43–55]. When these new studies were incorporated in the present update, the pooled hazards ratios of both OS and DFS/PFS/RFS were similar to those in the previous meta-analysis. Subgroup analyses based on two common confounders, tumor stage and MMR status, showed a significantly shorter OS for CIMP-H CRC patients with stages III-IV and pMMR tumors.

Changes in epigenetic modifications, especially DNA methylation status, are considered to be associated with the development and progression of CRC from early to advanced stages [8, 10]. What is particularly noteworthy is that genes (e.g., p16) methylated at an early stage in colorectal cancer might be demethylated due to ischemic conditions at later stages [8, 56]. These findings suggested that the prognostic role of CIMP might be variable among different tumor stages. However, based on stratified analyses according to tumor stage, different studies draw distinct or even contrary conclusions [13, 35, 36]. Thus, a subgroup meta-analysis was performed in our updated analysis to address this issue. We found that CIMP-H could increase the overall mortality risk by 1.52 times in stages III-IV CRC compared to CIMP-L/N, while in stages I-II CRC, no significant difference was observed in the overall survival of either group.

MMR status was also considered a notable factor affecting the prognostic value of CIMP and CRC. Due to limited studies included in the previous meta-analysis by Juo et al. [27], a subgroup analysis was only conducted for pMMR CRC patients. In the present update, we included enough qualified studies and were able to carry out a subset analysis for both pMMR and dMMR CRC patients. In a previous meta-analysis, an overall survival disadvantage was observed in CIMP-H/pMMR CRC patients. In contrast, this disadvantage did not hold true among the dMMR patients in our studies. The adverse prognosis from CIMP-H might be reversed by the favorable prognostic implication of dMMR among CRC patients [51, 57].

In addition to the prognostic value, the role of CIMP in predicting chemotherapy efficacy is another issue that needs to be addressed. Adjuvant chemotherapy is recommended as the standard therapy for locally advanced CRC; however, it does not benefit everyone. Thus, there is an urgent need to find useful biomarkers that can predict tumor chemosensitivity and response. CRC with different CIMP statuses has a unique gene expression profile [58]. This suggests that the expression level of genes related to drug transporters, drug receptors, drug-metabolizing enzymes, or other genes correlated with the pharmacokinetics of chemotherapeutic agents might be disparate between CIMP-H and CIMP-L/N CRCs, resulting in different chemosensitivities among patients. More importantly, CIMP has indeed been reported as a potential predictive biomarker for medication decisions, whereas results regarding the influence of CIMP on the efficacy of adjuvant chemotherapy were inconsistent [12, 24, 31, 35, 36, 50]. In this review, we conducted a subgroup analysis stratified by adjuvant chemotherapy, but we found no significant protective effect of adjuvant chemotherapy on DFS in either CIMP-H or CIMP-L/N CRC patients. Since only 3 studies with small sample sizes were included in this subgroup analysis, the statistical power of this result is obviously insufficient. An increasing number of clinical studies are required to determine whether CIMP can serve as a therapeutic biomarker.

The first limitation of this meta-analysis was that studies evaluating tumor progression by different indices, such as DFS, PFS, or RFS, were all included. To include as much relevant data as possible, we decided to use DFS, PFS, and RFS synonymously and combined them to estimate the prognostic value of CIMP-H. Although the proportion of patients with secondary primary cancer was small, it cannot be denied that the risk of bias could be inevitably introduced to this review. The second one was language bias due to the search conditions, which was limited to original English papers. The third limitation was the raw data bias due to data extraction and processing from the original literature when the HR and its 95% CI were not reported.

## 5. Conclusions

In conclusion, our meta-analysis updated some important evidence and confirmed that CIMP-H CRC had poorer OS and DFS/PFS/RFS than CIMP-L/N CRC. Additionally, the survival disadvantage of OS was observed particularly in stage III-IV and pMMR tumors. What's more, compared with surgery alone, surgery plus chemotherapy might not improve DFS outcomes for either CIMP-H or CIMP-L/N CRC patients. Additional studies with larger samples are required to provide further predictive information for the patient's quality of life.

## Figures and Tables

**Table 1:** Main characteristics of the included studies.

Author	Year	No. of patients	Continent/country	Age (years) median/mean, range	Median follow-up time (months)	Tumor	Stage	Treatment modality (%)	Assessment of CIMP				Outcome
									CIMP panel	Lab method	CIMP-H threshold	CIMP-H prevalence (%)	
Chang SC	2020	92	China	71.5	NR	CRC	I–IV	NR	Weisenberger+3^†^	MethyLight	≥5/8	25	OS
Chen KH	2019	450	China	NR	NR	CRC	I–IV	NR	Classic panel^‡^	MethyLight	≥3/5	16.4	OS
Kim SH	2018	194	Korea	62 (22−94)	38	CRC	II–IV	Adjuvant chemotherapy (100)	Classic panel +5^‡^	Pyrosequencing	≥3/10	33	OS
Gallois C	2018	1,867	France	NR	CIMP-H 73.2 CIMP-L 79.2	Colon	III	OX-based (100)	Weisenberger^†^	MSP	≥3/5	14.7	OS, DFS
Vedeld HM	2017	1118	Norway	72 (27-97)	NR	CRC	I–IV	NR	Weisenberger^†^	MSP	≥3/5	18	OS
Kim CH	2017	157	Korea	65 (31–88)	64.5	CRC	I–IV	NR	Weisenberger^†^	MSP	≥4/5	31.8	DFS
Bae JM	2017	950	Korea	CIMP-H 58 (36–72) CIMP-L 60 (24–80)	60.9	CRC	II–III	OX-based (100)	Weisenberger+3^†^	MethyLight	≥6/8	5.2	RFS
Lee MS	2016	198	USA	62 (29-94)	NR	CRC	I–IV	Anti-EGFR therapy (87.4)	Classic panel +1^‡^	Methylation450 bead-Chip	≥3/6	29.1	OS, PFS
Jia M	2016	1385	Germany	NR	58.8	CRC	I–IV	Surgery alone (54.3) surgery plus chemotherapy (45.4)	Other	MSP	≥3/5	13.6	OS
Cohen SA	2016	293	Greece	NR	74.5	CRC	II–III	OX-based (100)	Weisenberger^†^	MethyLight	≥3/5	9.6	OS, DFS
Cha Y	2016	153	Korea	61 (22-80)	41.8	CRC	I–IV	OX-based (83.7)	Weisenberger+3^†^	MethyLight	≥5/8	4.6	OS
Lee DW	2015	497	Korea	NR	65	CRC	II–III	OX-based (100)	Weisenberger+3^†^	MethyLight	≥5/8	5.8	OS, DFS
Kang KJ	2015	154	Korea	CIMP-H 61 (41–79) CIMP-L 60 (34–85)	60	Colon	I–IV	NR	Weisenberger^†^	MethyLight	≥3/5	17.5	RFS
Wang Y	2014	50	China	CIMP-H 57.8 CIMP-L 53.5	NR	CRC	II–III	OX-based (72)	Other	MSP	≥3/5	24	OS, DFS
Shiovitz S	2014	615	USA	63 (24-85)	57.6	Colon	III	5-FU based (100)	Weisenberger^†^	MethyLight	≥3/5	23	OS
Li X	2014	282	China	58.8 (25-81)	53	CRC	I–IV	5-FU based (NR)	Other	MS-HRM	≥4/7	13.12	OS
Hokazono K	2014	104	Japan	63.4	60	CRC	0-IV	Surgery alone (33.7) surgery plus chemotherapy (66.3)	Other	MSP	≥7/15	18.3	OS, RFS
Samadder NJ	2013	563	USA	73.9	NR	CRC	I–IV	NR	Weisenberger^†^	MethyLight	≥3/5	30	OS
Donada M	2013	120	Italy	67.6	112.8	Colon	II	Surgery alone (50) surgery plus 5-FU based (50)	Weisenberger^†^	MSP	≥3/5	18.3	OS, DFS
Bae JM	2013	734	Korea	62 (20–90)	56.6	CRC	I–IV	NR	Weisenberger+3^†^	MethyLight	≥5/8	6.4	OS, DFS
Rhee YY	2012	207	Korea	NR	46	CRC	I–IV	NR	Weisenberger+3^†^	MethyLight	≥5/8	30	OS
Kakar S	2012	33	USA	NR	NR	CRC	I–IV	NR	Other	MSP	≥3/7	48.5	OS
Jo P	2012	150	Germany	CIMP-H 62.7 CIMP-L 59.3	NR	Rectal	NR	5-FU based (100)	Weisenberger^†^	MSP	≥3/5	10	OS, DFS
Min BH	2011	245	Korea	65.0 (33–83)	44.5	CRC	I–IV	Adjuvant chemotherapy (100)	Weisenberger^†^	MethyLight	≥3/5	13.9	RFS
Ju HX	2011	78	Japan	Stage I–III 64.7 stage IV 62.1	NR	CRC	I–IV	NR	Classic panel^‡^	MSP	≥2/5	24.4	RFS
Jover R	2011	196	Spain	CIMP-H 73.7 CIMP-L 69.7	50.7	CRC	II–III	Surgery alone (48) surgery plus 5-FU based (52)	Other	Pyrosequencing	≥3/5	32.6	DFS
Sanchez JA	2009	391	USA	66.7	38.5	CRC	I–IV	NR	Weisenberger^†^	MethyLight	≥3/5	21.2	OS
Samowitz WS	2009	990	USA	NR	NR	Rectal	I–IV	NR	Classic panel^‡^	MSP	≥2/5	13.5	OS
Ogino S	2009	649	USA	66.5	NR	Colon	I–IV	NR	Weisenberger+3^†^	MethyLight	≥6/8	19.4	OS
Kim JH	2009	320	Korea	60.9	63.5	CRC	I–IV	NR	Weisenberger+3^†^	MethyLight	≥5/8	11.6	OS
Kalady MF	2009	357	USA	66.9	39.5	CRC	I–IV	NR	Weisenberger^†^	MethyLight	≥3/5	21.8	DFS
Lee S	2008	134	Korea	NR	40	Colon	I–IV	NR	Classic panel^‡^	MSP	≥2/5	31.3	OS
Kakar S	2008	69	USA	NR	NR, ≥60	CRC	I–IV	NR	Other	MSP	≥3/7	23.2	OS
Barault L	2008	582	France	NR	NR	Colon	I–IV	NR	Classic panel^‡^	MSP	≥4/5	16.7	OS
Shen L	2007	182	USA	NR	NR, >60	CRC	NR	NR	Classic panel +1^‡^	MSP, COBRA	≥2/6	15.4	OS
Samowitz WS	2005	756	USA	NR	65	Colon	I–IV	NR	Classic panel^‡^	MSP	≥2/5	24.7	OS

*Abbreviations:* No. = number; NR = not reported; 5-FU=fluorouracil; OX = oxaliplatin; MSP = methylation-specific PCR; COBRA = combined bisulfite restriction analysis; MS-HRM = methylation sensitive high resolution melting, OS = overall survival; PFS=progression-free survival; DFS=disease-free survival; RFS = recurrence-free survival. ^†^Weisenberger panel including 5 CIMP markers: CACNA1G, IGF2, NEUROG1, RUNX3, and SOCS1; Weisenberger+3 panel including 5 Weisenberger markers and CRABP1, MLH1, and p16. ^‡^Classic panel including 5 CIMP markers: MINT1, MINT2, MINT31, p16, and hMLH1; classic panel+1 including 5 classic markers and p14.; classic panel+5 including 5 classic markers and p14, DKK3, WNT5A, AXIN2, and TFAP2E.

## Data Availability

No data were used to support this study.
