# Supplementary material for: Meta-Analysis of the Prognostic and Predictive Role of the CpG Island Methylator Phenotype in Colorectal Cancer
Source: Dis Markers. 2022 Sep 15;2022:4254862. doi: 10.1155/2022/4254862 (PMC9499813; doi:10.1155/2022/4254862)

Supplementary appendix 1：PubMed Search Strategy

Search	Query	Items found
#1	"Colorectal Neoplasms"[MeSH Terms]	211,700
#2	"neoplasms colorectal"[Title/Abstract] OR "colorectal neoplasm"[Title/Abstract] OR "neoplasm colorectal"[Title/Abstract] OR "colorectal tumors"[Title/Abstract] OR "colorectal tumor"[Title/Abstract] OR "tumor colorectal"[Title/Abstract] OR "tumors colorectal"[Title/Abstract] OR "colorectal carcinoma"[Title/Abstract] OR "carcinoma colorectal"[Title/Abstract] OR "carcinomas colorectal"[Title/Abstract] OR "colorectal carcinomas"[Title/Abstract] OR "colorectal cancer"[Title/Abstract] OR "cancer colorectal"[Title/Abstract] OR "cancers colorectal"[Title/Abstract] OR "colorectal cancers"[Title/Abstract]	126,916
#3	Search (#1) OR #2	247,832
#4	"cpg island methylator phenotype"[Title/Abstract] OR "CIMP"[Title/Abstract]	1,212
#5	"Predictive"[Title/Abstract] OR "Predictor"[Title/Abstract] OR "prognosis"[Title/Abstract] OR "prognostic"[Title/Abstract] OR "survival"[Title/Abstract]	1,851,055
#6	Search (((#3) and #4) and #5	333

Supplementary appendix 2：Quality Assessment of Included Studies

NEWCASTLE - OTTAWA QUALITY ASSESSMENT SCALE
COHORT STUDIES

Selection

1) Representativeness of the exposed cohort

a) truly representative of the average "colorectal cancer patient with CIMP-high" in the community*

b) somewhat representative of the average "colorectal cancer patient with CIMP-high"in the community*

c) selected group of users, e.g., nurses, volunteers

d) no description of the derivation of the cohort

2) Selection of the non-exposed cohort "colorectal cancer patient with CIMP-low or CIMP-negative"

a) drawn from the same community as the exposed cohort *****

b) drawn from a different source

c) no description of the derivation of the non exposed cohort

3) Ascertainment of exposure

a) secure record (e.g., surgical records)*****

b) structured interview *****

c) written self report

d) no description

4) Demonstration that outcome of interest was not present at start of study

a) yes*****

b) no

Comparability

1) Comparability of cohorts on the basis of the design or analysis

a) study controls for "presence or absence of CIMP-high" *

b) study controls for any additional factor (age, sex, stage, primary tumor location) *

Outcome

1) Assessment of outcome (death or tumor progression)

a) independent blind assessment *****

b) record linkage *****

c) self report

d) no description

2) Was follow-up long enough for outcomes to occur

a) yes (2 years) *****

b) no

3) Adequacy of follow up of cohorts

a) complete follow up - all subjects accounted for *****

b) subjects lost to follow up unlikely to introduce bias - small number lost (<25%) or description of those lost *

c) follow up rate < 75% and no description of those lost

d) no statement

Author	Selection	Comparability	Outcome	Author	Selection	Comparability	Outcome
**Chang SC**	****	*	**	**Donada M**	****	*	***
**Chen KH**	****	*	**	**Bae JM**	****	*	***
**Kim SH**	****	**	***	**Rhee YY**	****	**	***
**Gallois C**	****	**	***	**Kakar S**	****	*	**
**Vedeld HM**	****	**	**	**Jo P**	****	*	**
**Kim CH**	****	**	***	**Min BH**	****	*	***
**Bae JM**	****	**	***	**Ju HX**	****	*	**
**Lee MS**	****	**	**	**Jover R**	****	**	***
**Jia M**	****	**	***	**Sanchez JA**	****	**	***
**Cohen SA**	****	*	***	**Samowitz WS**	****	**	**
**Cha Y**	****	**	***	**Ogino S**	****	**	**
**Lee DW**	****	*	***	**Kim JH**	****	*	***
**Kang KJ**	****	**	***	**Kalady MF**	****	**	***
**Wang Y**	****	**	**	**Lee S**	****	*	***
**Shiovitz S**	****	*	***	**Kakar S**	****	**	***
**Li X**	****	**	***	**Barault L**	****	**	**
**Hokazono K**	****	*	***	**Shen L**	****	**	***
**Samadder NJ**	****	**	**	**Samowitz WS**	****	*	***

Supplementary appendix 3： Supplementary Figures

Supplementary Figure 1. Funnel plot of (A) overall survival (OS) for estimation of publication bias.

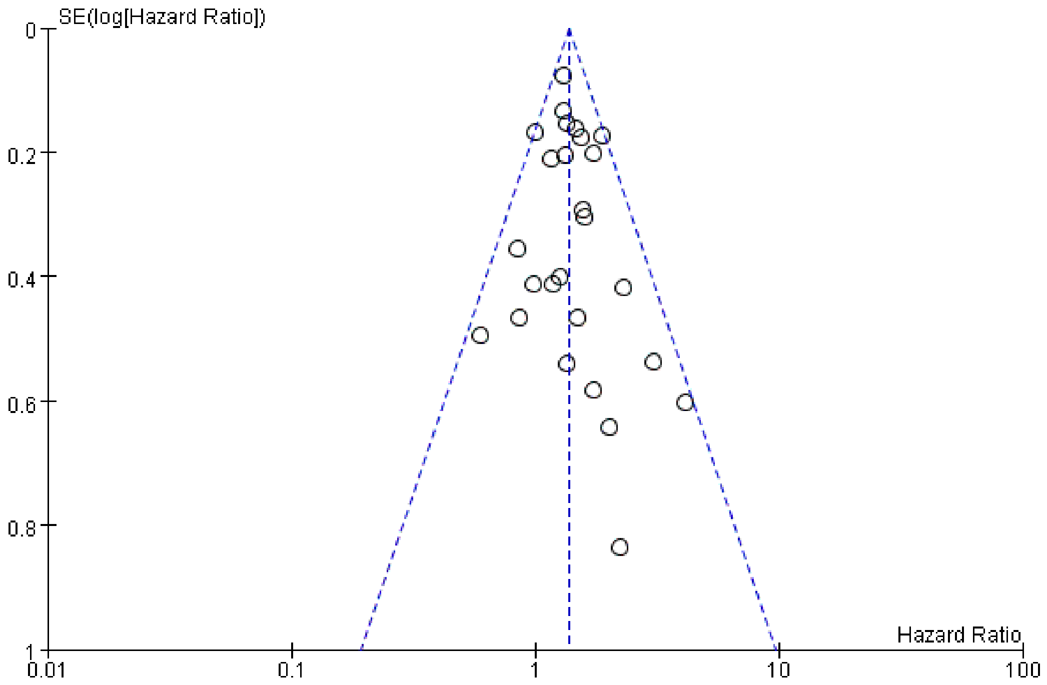

Supplementary Figure 2. Sensitivity analysis for the effect of each study on the pooled hazard ratios (HRs) of overall survival (OS).

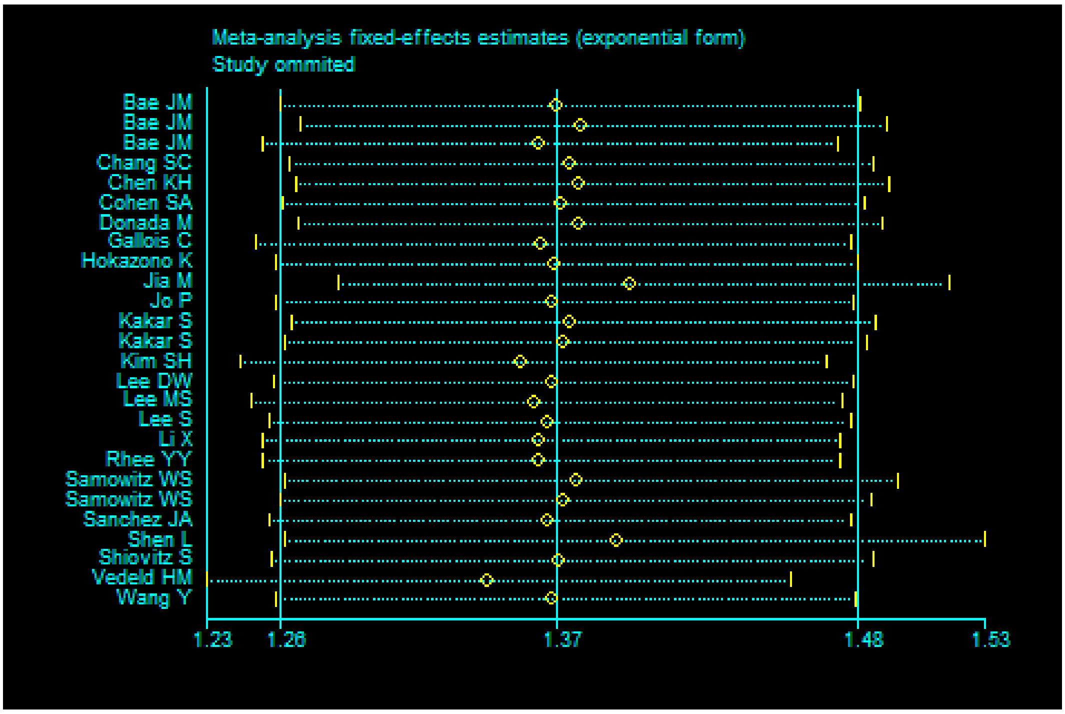

Supplementary Figure 3. Forest plots of HRs of OS in studies of colorectal cancer (CRC) patients associated with CIMP with restriction to tumor stage.

Study or Subgroup	log[Hazard Ratio]	SE	Weight	Hazard Ratio IV, Random, 95% CI
**2.2.1 stage I-II**				
Cohen SA 2016	0.122218	1.072354	5.3%	1.13 [0.14, 9.24]
Donada M 2013	-0.51083	0.493189	24.9%	0.60 [0.23, 1.58]
Jia M 2016	-0.34249	0.320565	58.9%	0.71 [0.38, 1.33]
Li X 2014	-0.65393	0.744329	10.9%	0.52 [0.12, 2.24]
**Subtotal (95% CI)**			**100.0%**	**0.67 [0.42, 1.09]**
Heterogeneity: Tau² = 0.00; Chi² = 0.44, df = 3 (P = 0.93); I² = 0%				
Test for overall effect: Z = 1.60 (P = 0.11)				
**2.2.2 stage III-IV**				
Chen KH 2019	1.722767	0.495545	2.9%	5.60 [2.12, 14.79]
Cohen SA 2016	0.371564	0.469947	3.2%	1.45 [0.58, 3.64]
Gallois C 2018	0.378436	0.164001	14.4%	1.46 [1.06, 2.01]
Jia M 2016-III	0.262364	0.314322	6.3%	1.30 [0.70, 2.41]
Jia M 2016-IV	0.139762	0.263712	8.2%	1.15 [0.69, 1.93]
Li X 2014	0.559616	0.312188	6.4%	1.75 [0.95, 3.23]
Shen L 2007	0.270027	0.07856	22.9%	1.31 [1.12, 1.53]
Shiovitz S 2014	0.307485	0.153014	15.4%	1.36 [1.01, 1.84]
Vedeld HM 2017-III	0.378436	0.204365	11.4%	1.46 [0.98, 2.18]
Vedeld HM 2017-IV	0.940007	0.247188	8.9%	2.56 [1.58, 4.16]
**Subtotal (95% CI)**			**100.0%**	**1.52 [1.27, 1.81]**
Heterogeneity: Tau² = 0.03; Chi² = 15.70, df = 9 (P = 0.07); I² = 43%				
Test for overall effect: Z = 4.65 (P < 0.00001)				

Test for subgroup differences: Chi² = 9.58, df = 1 (P = 0.002), I² = 89.6%

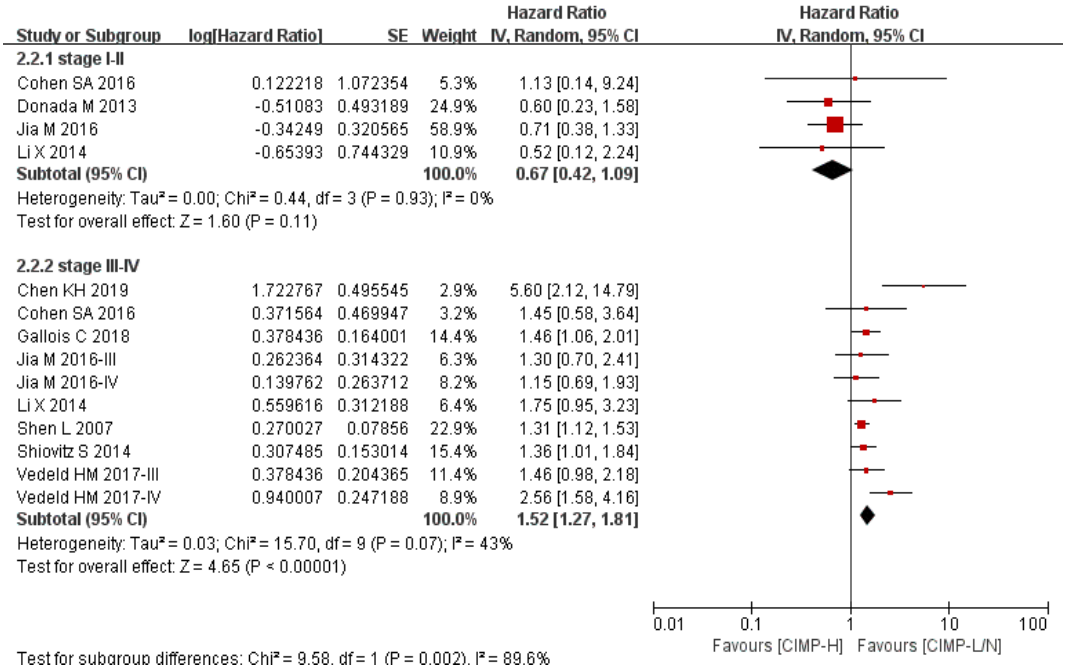

Supplementary Figure 4. Forest plots of HRs of OS in studies of colorectal cancer (CRC) patients associated with CIMP with restriction to MMR status.

Study or Subgroup	log[Odds Ratio]	SE	Weight	Odds Ratio IV, Random, 95% CI
**2.3.1 pMMR**				
Cohen SA 2016	0.378436	0.433384	5.9%	1.46 [0.62, 3.41]
Gallois C 2018	0.652325	0.138226	16.8%	1.92 [1.46, 2.52]
Jia M 2016	0.019803	0.18391	14.4%	1.02 [0.71, 1.46]
Kakar S 2008	-0.15315	0.464849	5.3%	0.86 [0.34, 2.13]
Lee S 2008	0.672944	0.312612	9.0%	1.96 [1.06, 3.62]
Ogino S 2009	-0.47804	0.306385	9.2%	0.62 [0.34, 1.13]
Samowitz WS 2005	0.262364	0.133859	17.0%	1.30 [1.00, 1.69]
Sanchez JA 2009	0.506818	0.347499	7.9%	1.66 [0.84, 3.28]
Vedeld HM 2017	0.609766	0.183705	14.4%	1.84 [1.28, 2.64]
**Subtotal (95% CI)**			**100.0%**	**1.37 [1.08, 1.75]**
Heterogeneity: Tau² = 0.07; Chi² = 20.16, df = 8 (P = 0.010); I² = 60%				
Test for overall effect: Z = 2.55 (P = 0.01)				
**2.3.2 dMMR**				
Chang SC 2020	-0.01005	0.41288	14.7%	0.99 [0.44, 2.22]
Cohen SA 2016	-0.17435	1.128278	4.5%	0.84 [0.09, 7.67]
Gallois C 2018	0.09531	0.340561	16.5%	1.10 [0.56, 2.14]
Jia M 2016	-0.06188	0.043527	22.4%	0.94 [0.86, 1.02]
Ogino S 2009	1.252763	0.34162	16.5%	3.50 [1.79, 6.84]
Rhee YY 2012	1.115142	0.535486	11.9%	3.05 [1.07, 8.71]
Sanchez JA 2009	1.156881	0.464476	13.5%	3.18 [1.28, 7.90]
**Subtotal (95% CI)**			**100.0%**	**1.63 [0.96, 2.76]**
Heterogeneity: Tau² = 0.32; Chi² = 25.85, df = 6 (P = 0.0002); I² = 77%				
Test for overall effect: Z = 1.81 (P = 0.07)				

Test for subgroup differences: Chi² = 0.34, df = 1 (P = 0.56), I² = 0%

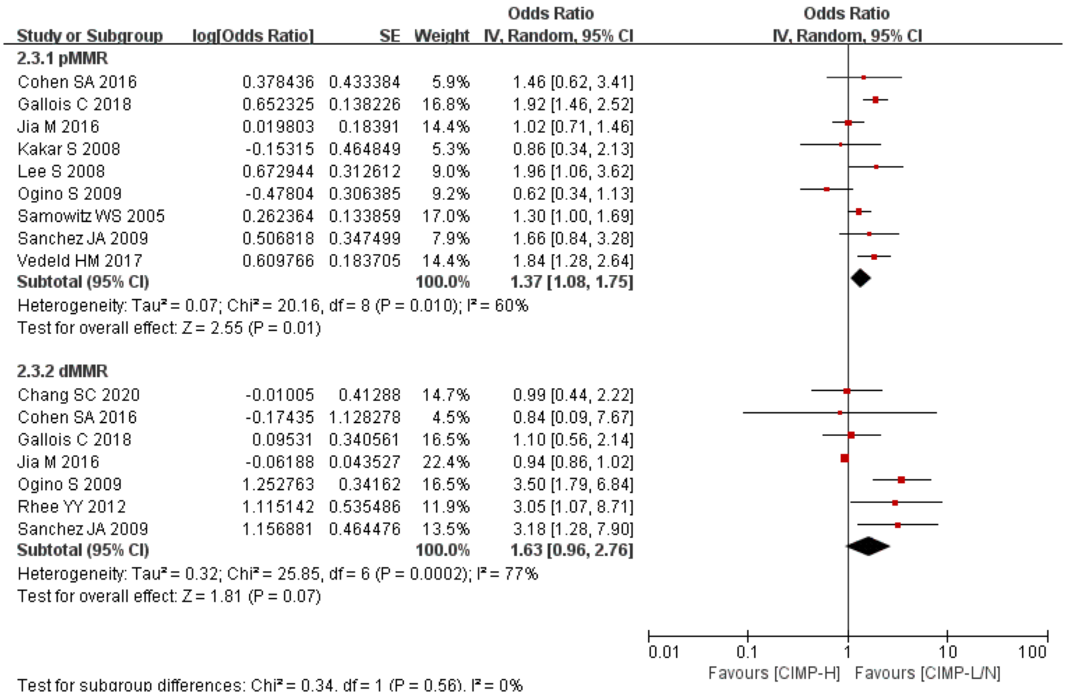

Supplementary Figure 5. Funnel plot of disease-free survival/progression-free survival /recurrence-free survival (DFS/PFS/RFS) for estimation of publication bias.

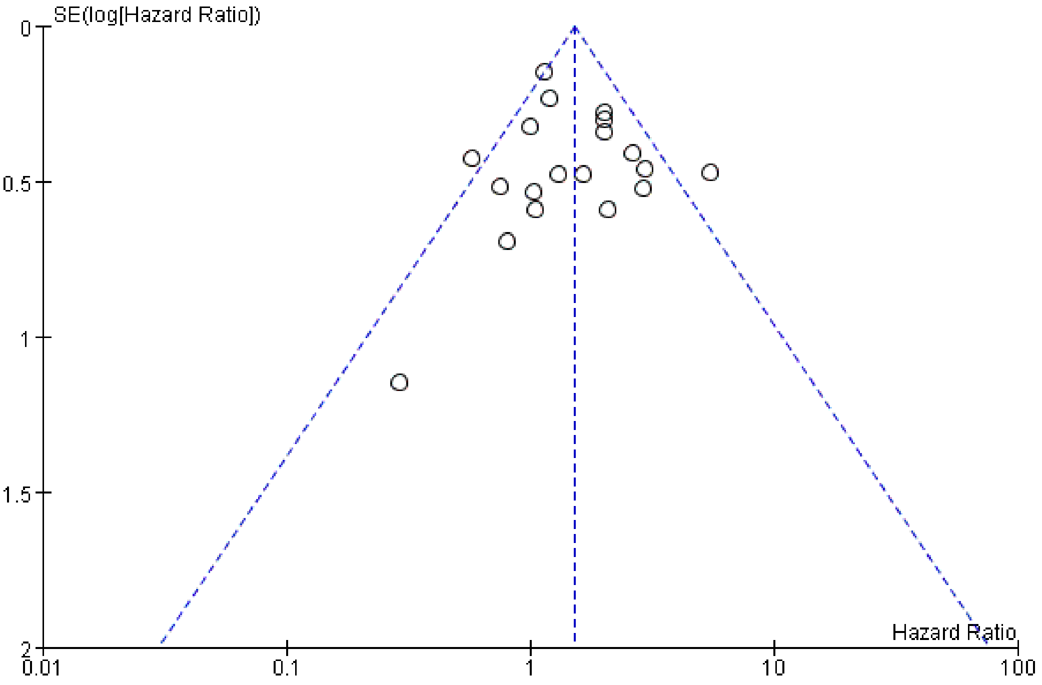

Supplementary Figure 6. Sensitivity analysis for the effect of each study on the pooled hazard ratios (HRs) of disease-free survival/progression-free survival /recurrence-free survival (DFS/PFS/RFS).

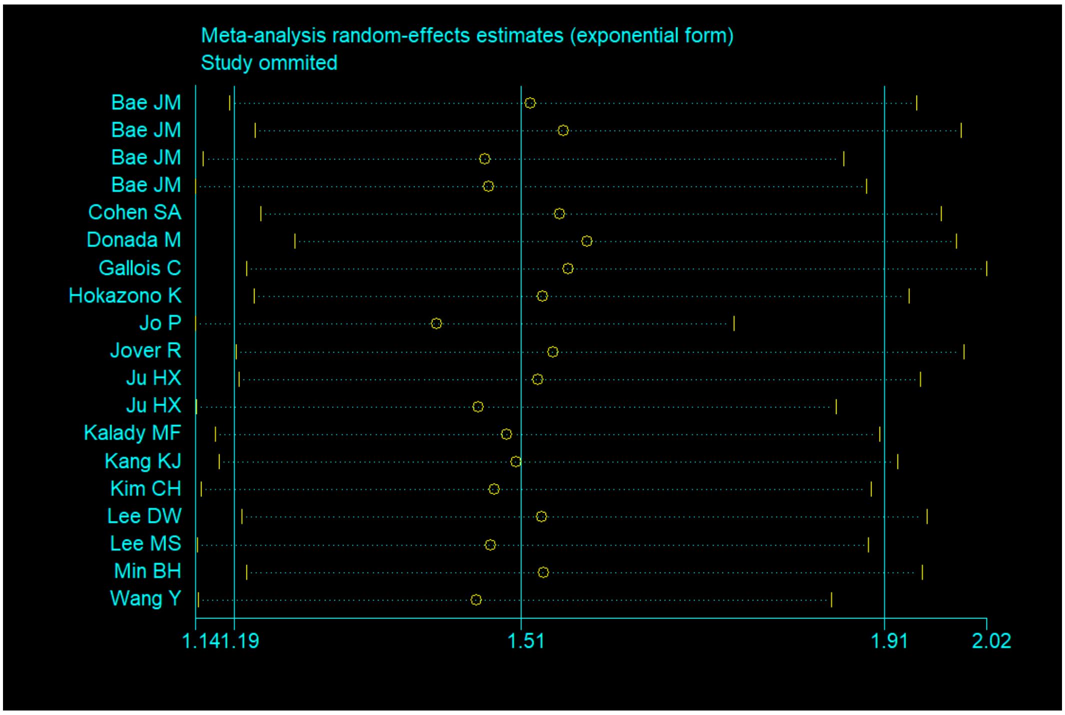

Supplementary Figure 7. Forest plots of HRs of OS in studies of colorectal cancer (CRC) patients associated with CIMP stratified by laboratory methods.

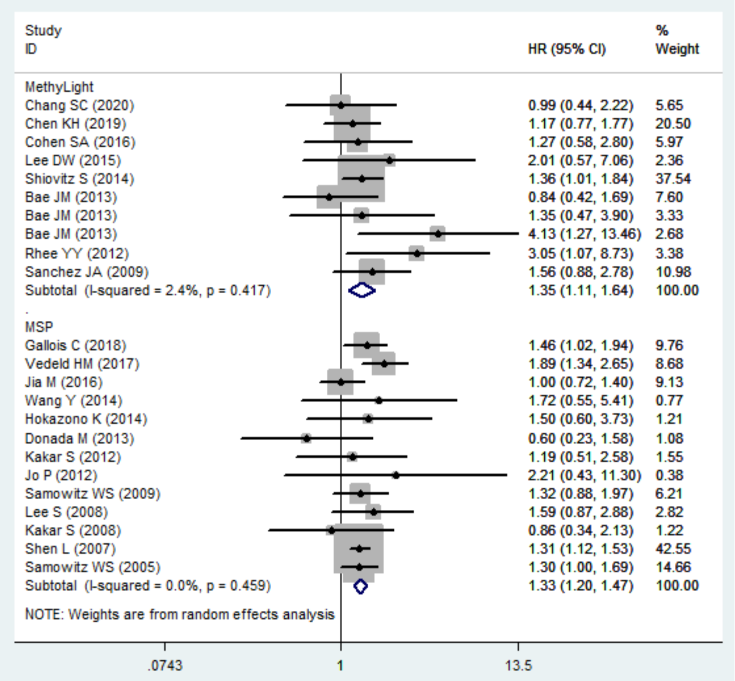

Supplementary Figure 8. Forest plots of HRs of OS in studies of colorectal cancer (CRC) patients associated with CIMP stratified by gene panels.

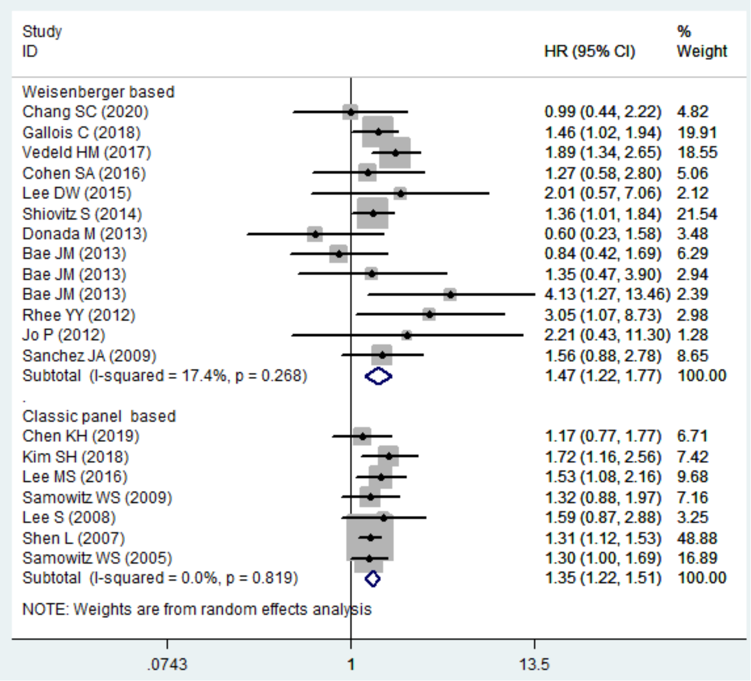

Supplement: Supplementary Materials — Supplementary data associated with this article can be found in the online version. [file 4254862.f1.zip › 4254862.f1/SUPPLEMENTARY MATERIAL (1).pdf]
